# Recovery of Iron, Silver and Lead from Zinc Ferrite Residue

**DOI:** 10.3390/ma18153522

**Published:** 2025-07-27

**Authors:** Peter Iliev, Biserka Lucheva, Nadezhda Kazakova, Vladislava Stefanova

**Affiliations:** Department of Non-Ferrous Metals and Alloys, Faculty of Metallurgy and Materials Science, University of Chemical Technology and Metallurgy, 1756 Sofia, Bulgaria; plasma@uctm.edu (B.L.); n_kazakova@uctm.edu (N.K.); vps@uctm.edu (V.S.)

**Keywords:** zinc ferrite residue, sulfuric acid leaching, chloride leaching

## Abstract

The present article aims to develop a technological scheme for processing zinc ferrite residue, which typically forms during the leaching of zinc calcine. This semi-product is currently processed through the Waelz process, the main disadvantage of which is the loss of precious metals with the Waelz clinker. The experimental results of numerous experiments and analyses have verified a technological scheme including the following operations: sulfuric acid leaching of zinc ferrite residue under atmospheric conditions; autoclave purification of the resulting productive solution to obtain hematite; chloride leaching of lead and silver from the insoluble residue, which was produced in the initial operation; and cementation with zinc powder of lead and silver from the chloride solution. Utilizing such an advanced methodology, the degree of zinc leaching is 98.30% at a sulfuric acid concentration of 200 g/L, with a solid-to-liquid ratio of 1:10 and a temperature of 90 °C. Under these conditions, 96.40% Cu and 92.72% Fe form a solution. Trivalent iron in the presence of seeds at a temperature of 200 °C precipitates as hematite. In chloride extraction with 250 g/L NaCl, 1 M HCl, and a temperature of 60 °C, the leaching degree of lead is 96.79%, while that of silver is 84.55%. In the process of cementation with zinc powder, the degree of extraction of lead and silver in the cement precipitate is 98.72% and 97.27%, respectively. When implementing this scheme, approximately 15% of the insoluble residue remains, containing 1.6% Pb and 0.016% Ag.

## 1. Introduction

The treatment of zinc ferrite residues (ZFRs), generated during the acid leaching of zinc calcine, represents a critical stage for both the efficient recovery of non-ferrous metals and the mitigation of associated environmental risks. The primary phase in these residues —zinc ferrite (ZnFe_2_O_4_)—is a chemically stable spinel compound, exhibiting low solubility under conventional hydrometallurgical conditions [1,2]. Therefore, overcoming its chemical inertness requires the application of various technological approaches, which are generally classified into four main groups: pyrometallurgical, combined (roasting–leaching), hydrometallurgical (including high-pressure processes), and bioleaching methods.

Pyrometallurgical processing of ZFR involves the high-temperature reduction of ZnFe_2_O_4_ followed by the capture of volatilized zinc. The most widely used method is the Waelz process [2,3,4], in which a mixture of ZFR and a carbonaceous reductant (such as coal or coke) is treated in a rotary kiln at temperatures between 1000 and 1250 °C. Under these conditions, zinc is reduced and volatilized, then oxidized and captured as ZnO from the off-gases. Iron remains in the slag as stable oxides. Zinc recovery typically ranges between 85% and 90%. Industrial Waelz kilns are operated by companies such as Zhuzhou Smelter (China), Votorantim (Brazil), Glencore (Italy), and KCM Plovdiv (Bulgaria) [5].

An alternative pyrometallurgical method is the Top-Submerged Lance (TSL) process, in which fine residues are injected directly into molten slag [6,7]. The intense mixing and high temperature promote rapid reduction and volatilization of zinc. The Ausmelt technology, based on this principle, is applied at plants in Whyalla (Australia), Onsan, and Sukpo (South Korea) [5]. This process achieves recoveries of approximately 82% zinc, 92% lead, 86% silver, and 61% copper from the residues [7].

Combined pyro-hydrometallurgical technologies for processing ZFR typically involve roasting followed by leaching, aiming to convert zinc into water-soluble forms. In reductive roasting, ZnFe_2_O_4_ is transformed into ZnO and magnetite, after which acid leaching is applied. Laboratory tests conducted at 750 °C for 90 min, with 8% CO and 90 g/L H_2_SO_4_, achieved recoveries of 61.38% zinc and 80.9% iron [8]. Sulfatizing roasting using iron sulfate at 640 °C for 30 min leads to the conversion of various zinc forms into ZnSO_4_, achieving up to 90.9% zinc recovery with minimal soluble iron [9,10]. Roasting with ammonium sulfate in a three-step process results in the formation of zinc and iron sulfates, enabling selective metal extraction [11]. Combined sulfatizing roasting and acid leaching (200 °C, 1 h, H_2_SO_4_:solid = 1:1) can yield up to 90% zinc extraction. Under milder conditions (95 °C, 2 h, 200 g/L H_2_SO_4_), 51% recovery of ferritic zinc and 82% total zinc have been reported [12,13].

Hydrometallurgical technologies can be performed at atmospheric or elevated pressure. The most common method is hot sulfuric acid leaching followed by iron precipitation as jarosite, goethite, or hematite. This achieves high zinc and iron yields but makes impurity removal challenging [14]. Reductive leaching improves extraction of zinc, iron, and indium by reducing Fe^3+^ to Fe^2+^, simplifying downstream processing. Reductants such as sphalerite, galena, and sulfur dioxide are effective in lab studies [14,15,16]. For example, leaching with sphalerite (0.95 theoretical amount) at 150 g/L H_2_SO_4_, 90 °C, for 4 h achieves 94.8% indium, 96.1% zinc, and 92.8% iron recovery [13]. Using galena under similar conditions yields 88% indium, 87% zinc and 91% iron [15]. Sulfur dioxide as reductant also effectively reduces Fe^3+^ to Fe^2+^, eliminating separation issues for indium [16,17].

Oxidative leaching with hydrogen peroxide or manganese dioxide lowers iron dissolution significantly while maintaining acceptable zinc extraction. Iron recovery drops from 70% (without oxidants) to 0.4% with MnO_2_ and 5% with H_2_O_2_, due to iron hydroxide or iron–manganese compound formation [18].

Alkaline leaching using NaOH or Na_2_CO_3_ at pH > 12 and 60–90 °C selectively dissolves zinc as zincates, leaving iron in the residue. However, this process is slower, with 70–85% yields and limited industrial use [19,20,21].

Pressure leaching (autoclave) uses elevated temperatures (up to 250 °C) and pressures (4–5 MPa) with oxygen or air to fully transform zinc ferrite into soluble zinc sulfate and insoluble iron precipitates. Extraction efficiency can exceed 95%, but costs are higher than atmospheric leaching [22,23].

Bioleaching employs bacteria like *Acidithiobacillus ferrooxidans* to oxidize iron and sulfur compounds, aiding zinc ferrite breakdown under acidic conditions. Though slower, this environmentally friendly method suits low-grade or finely dispersed residues and is under development worldwide [24].

Choosing the right technology depends on ZFR composition, metal content, and economic and environmental considerations. Pyrometallurgical methods offer robustness and high productivity, while hydrometallurgical and combined approaches provide flexibility and better integration with circular economy goals.

This study aims to contribute to the development of effective and environmentally sustainable strategies for the treatment of ZFR by exploring alternative leaching methods under both atmospheric and pressure conditions. The key technological breakthrough targeted in this work is the enhanced dissolution of the chemically stable ZnFe_2_O_4_ spinel phase through optimized leaching parameters and process design. By systematically evaluating the leaching efficiency, residue composition, and metal recovery, this study seeks to provide practical insights for improving non-ferrous metal extraction from complex secondary materials.

## 2. Materials and Methods

### 2.1. Materials

The initial ZFR was supplied by the Bulgarian zinc production plant KCM. The material was subjected to a water washing process aimed at removing water-soluble zinc, conducted at a temperature of 60 °C with a solid-to-liquid ratio of 1:4 and continuous mechanical stirring. After this treatment, the resulting residue was dried, ground, and homogenized using a knife mill. The chemical composition of the prepared material was subsequently determined using both conventional chemical methods and inductively coupled plasma optical emission spectroscopy (ICP-OES) with a Prodigy spectrometer. The results were summarized in Table 1.

**Figure 1:**
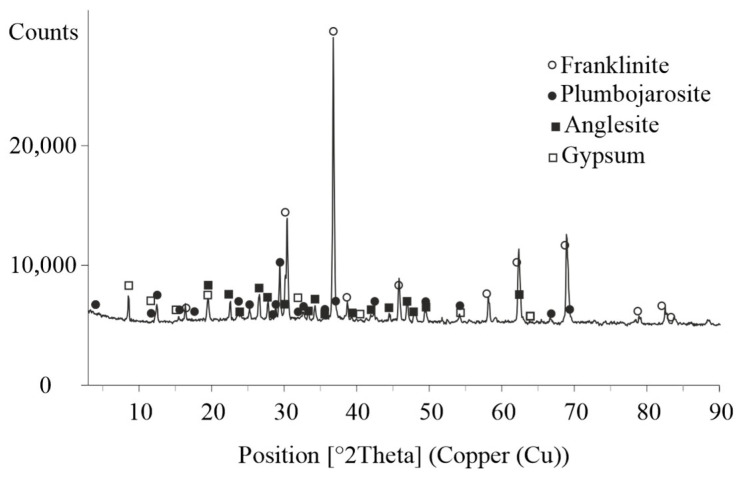
XRD pattern of the initial ZFR.

The XRD analysis (Figure 1) identified the main phase in the ZFR as the spinel type oxide franklinite (ZnFe_2_O_4_). This compound is formed during the roasting of zinc concentrates and persists in the insoluble residue following acid leaching of the zinc calcine due to its chemical inertness under the applied conditions. Additionally, plumbojarosite, an iron–lead sulfate mineral known for its stability and low solubility, was detected. The presence of anglesite (PbSO_4_), a stable lead sulfate formed either by the oxidation of galena (PbS) or by direct reaction between lead and sulfuric acid, was also confirmed. Furthermore, the diffractogram revealed gypsum (CaSO_4_·2H_2_O), which results from the reaction of sulfuric acid with calcium-containing compounds.

### 2.2. Leaching Experiments and Methods

The sulfuric acid leaching experiments of the ZFR under atmospheric conditions were performed in 1 L cylindrical reaction vessels (Lenz Laborglasinstrumente™, Wertheim, Germany), equipped with a thermostatic jacket and a withdrawal valve to maintain temperature control and facilitate sample extraction. For experiments conducted at elevated pressure, a 2 L glass autoclave (model TLA 30) was employed to ensure controlled pressure conditions during the leaching process.

The filtrate obtained under the optimal conditions of the sulfuric acid leaching was neutralized with a 10% solution of Ca(OH)_2_ to the desired pH value. The neutralized solution was used to perform studies on the autoclave precipitation of iron in the form of hematite. The experiments were carried out in a laboratory novoclave from Büchi AG, with a reactor (400 mL) constructed of stainless steel Hastelloy.

The chloride leaching experiments on lead cake were carried out using a 300 mL vessel in a thermostatically controlled water bath with mechanical stirring.

Upon completion of the experiments, the resulting pulp was filtered, and the insoluble residue was dried at 353 K for 24 h and subsequently weighed using an analytical balance. The concentrations of the target metals in both process insoluble residues were determined using inductively coupled plasma optical emission spectrometry (ICP-OES, Prodigy). The extraction degrees of the target metals were calculated based on the chemical composition of the insoluble residues remaining after the leaching process. The acidity of the leach solution at the end of each experiment was measured using a combined pH electrode Aqualytic SD300, produced by Aqualytic GmbH & Co., Dortmund, Germany.

The concentration of divalent iron in the solution was determined by redox titration using potassium permanganate, while the concentration of sulfuric acid was quantified via acid-base titration with sodium hydroxide.

The effects of temperature, process duration, reagent concentration, and solid-to-liquid ratio on the leaching degree of zinc, iron, copper, lead, and silver were systematically investigated during sulfuric acid leaching of ZFR and chloride leaching of lead cake. Additionally, in the autoclave precipitation of iron, the influence of temperature and the presence of seed crystals on the extent of hematite precipitation were examined.

The phase composition of the insoluble residues was determined by X-ray diffraction (XRD) using a Philips PW 1050 diffractometer (Philips Analytical, Eindhoven, Netherlands) equipped with Cu-Kα radiation (λ = 1.5406 Å), operating at 40 kV and 30 mA, with data collected over a 2θ range of 5° to 90°.

The morphology and phase composition of the ZFR and lead cakes were studied using a scanning electron microscope (SEM) from Carl Zeiss Microscopy GmbH, Oberkochen, Germany, coupled with Energy-Dispersive X-ray Spectroscopy (EDS) from Oxford Instruments. SEM and EDS analyses of the hematite and the residue after chloride leaching were performed using an X-MaxN 50 mm^2^ EDS detector (Oxford Instruments, Oxford, UK; 20 kV accelerating voltage), mounted on a Tescan Vega 3 XMU electron microscope (Tescan, Brno, Czech Republic).

## 3. Results and Discussions

### 3.1. Sulfuric Acid Leaching of ZFR

The ZFR was subjected to both atmospheric and autoclave leaching to evaluate the most suitable processing method. Sulfuric acid, a cost-effective and widely available reagent, serves as an efficient leaching agent for a broad spectrum of metals. In Bulgaria, sulfuric acid is industrially produced from metallurgical SO_2_ off-gases, which are generated during the processing of copper, lead, and zinc sulfide concentrates. The present study investigates the influence of key process parameters—namely temperature, sulfuric acid concentration, leaching duration, and solid-to-liquid ratio—on metal leaching degree and the phase composition of the resulting solid residues.

#### 3.1.1. Effect of Temperature

**Figure 2:**
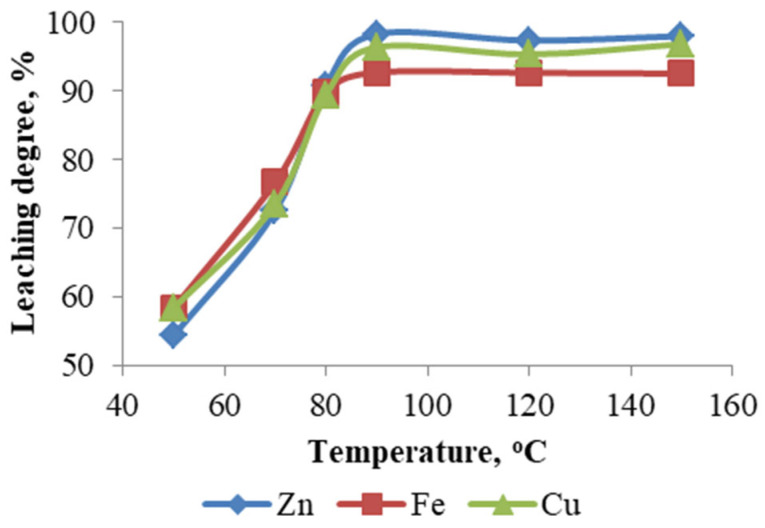
Effect of temperature.

The influence of temperature on the sulfuric acid leaching of ZFR was systematically studied within the range of 50 to 150 °C, at a fixed sulfuric acid concentration of 200 g/L, a solid-to-liquid ratio of 1:10, and a leaching duration of 3 h. The data presented in Figure 2 demonstrate a pronounced increase in the leaching efficiencies of zinc, iron, and copper with temperature elevation from 50 to 90 °C, achieving maximum extraction rates of 98.30%, 92.72%, and 96.40%, respectively, at 90 °C. Further temperature increases under autoclave conditions (120 and 150 °C) did not yield any significant enhancement in the extraction of the target metals, indicating that 90 °C represents the optimal temperature for efficient metal recovery under the studied conditions.

#### 3.1.2. Effect of Sulfuric Acid Concentration

**Figure 3:**
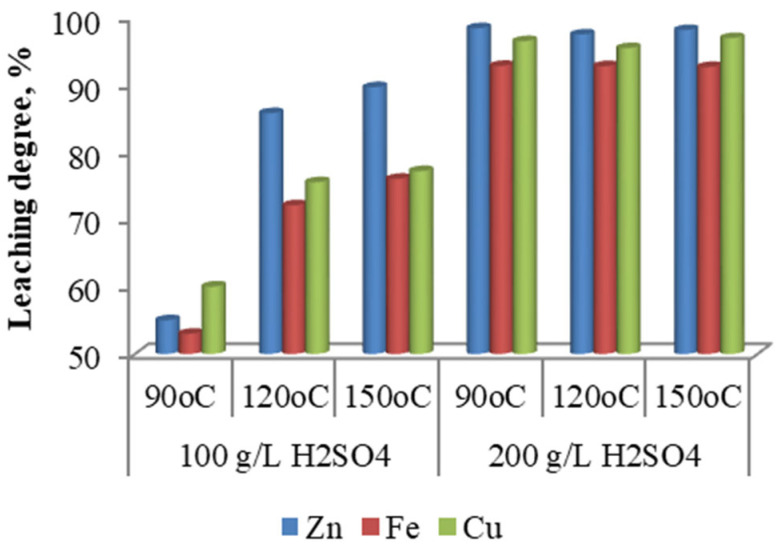
Effect of sulfuric acid concentration.

Experiments aimed at evaluating the influence of sulfuric acid concentration on the leaching process were conducted at initial acid concentrations of 100 g/L and 200 g/L, under three different temperatures—90, 120, and 150 °C—while maintaining all other process parameters constant. The experimental results (Figure 3) indicate that at an initial sulfuric acid concentration of 100 g/L, the extraction efficiencies of zinc, iron, and copper remain low across all temperatures, with particularly poor performance observed under atmospheric leaching at 90 °C. Increasing the initial acid concentration to 200 g/L led to a significant improvement in metal recovery across all studied temperatures, highlighting the critical role of acid concentration in enhancing leaching efficiency.

#### 3.1.3. Effect of the Solid-to-Liquid Ratio

The solid-to-liquid ratio is a key parameter influencing the mass transfer processes occurring during the interaction between the solid phase and the leaching solution. Laboratory experiments to assess the effect of the solid-to-liquid ratio on metal extraction efficiency were conducted at ratios of 1:10, 1:7, and 1:5, with a leaching duration of 3 h. Atmospheric leaching was carried out at 90 °C, while autoclave leaching of ZFR was performed at 150 °C.

**Figure 4:**
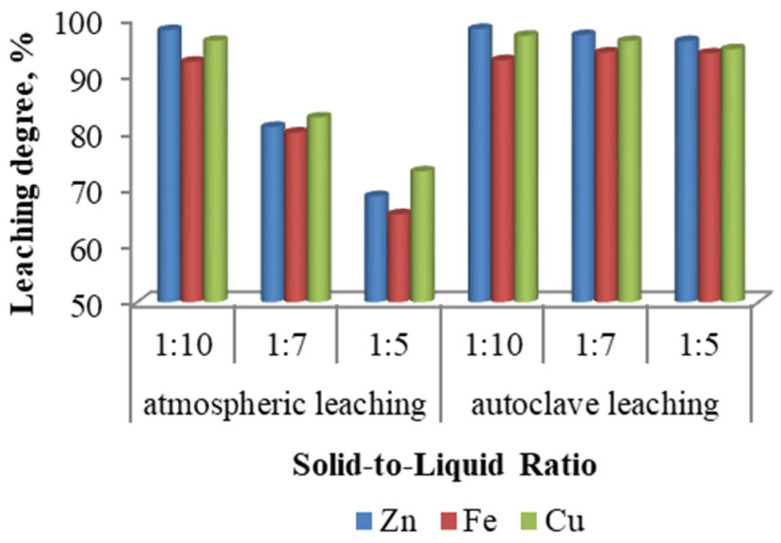
The effect of the solid-to-liquid ratio.

As illustrated in Figure 4, this parameter exerts a more pronounced influence under atmospheric leaching conditions, where a decrease in the solid-to-liquid ratio significantly enhances the extraction of zinc, iron, and copper. In contrast, under autoclave leaching conditions, increasing the solid content leads to only a slight reduction in extraction efficiencies. The optimal solid-to-liquid ratio for atmospheric leaching was found to be 1:10, at which the maximum leaching degrees for zinc, iron, and copper were achieved.

#### 3.1.4. Effect of Reaction Time

**Figure 5:**
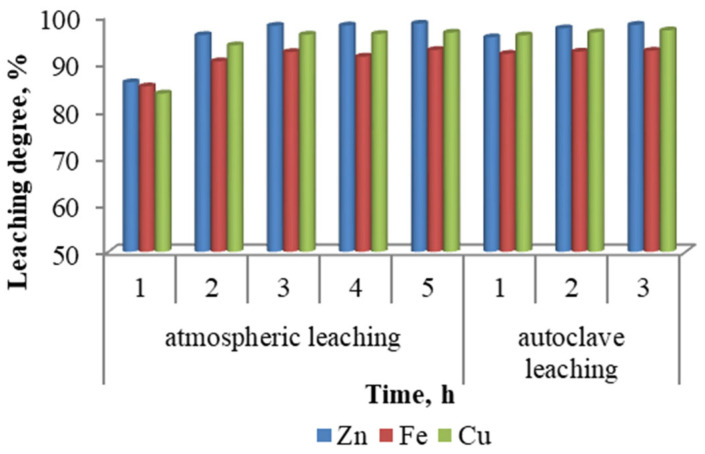
Effect of reaction time.

The duration of the leaching experiments conducted under atmospheric conditions extended up to 5 h, as shown in Figure 5, whereas the autoclave leaching tests were limited to a maximum duration of 3 h. Under atmospheric leaching, the metal extraction efficiencies increased markedly during the first 2 h, after which only a marginal improvement was observed with further extension of the leaching time. In contrast, during autoclave leaching, the most significant increase in metal recovery occurred within the first hour. Subsequent increases in leaching duration had little to no effect on the extraction efficiencies. These findings indicate that the leaching kinetics are substantially faster under autoclave conditions, and prolonged treatment times offer diminishing returns in terms of metal recovery.

Based on the conducted investigation of the sulfuric acid leaching process of ZFR under both atmospheric and autoclave conditions, the following key conclusions can be drawn:

- Sulfuric acid concentration is a critical factor determining the metal extraction efficiency. A minimum initial concentration of 200 g/L is required to achieve complete dissolution of copper, iron, and zinc.
- Temperature plays a major role in the leaching process. Practically complete leaching of the ZFR requires a minimum temperature of 90 °C.
- The solid-to-liquid ratio significantly affects metal extraction only under atmospheric leaching conditions, with lower ratios favoring higher extraction rates.
- The optimal leaching duration under atmospheric conditions is approximately 3 h while under autoclave conditions, 1 h is sufficient to achieve high extraction rates. Beyond this timeframe, only marginal improvements in metal recovery are observed.
- Autoclave leaching offers higher process rates and allows for operation at higher pulp densities, increasing overall efficiency. However, it requires specialized pressure-resistant equipment, which raises the cost of implementation.

These findings provide a solid basis for selecting optimal leaching conditions depending on available resources and desired process efficiency.

#### 3.1.5. Characterization of the Products of Sulfuric Acid Leaching

Table 2 presents the chemical composition of the leachate and the solid residue obtained under optimal conditions of atmospheric and autoclave sulfuric acid leaching of ZFR. The data showed that the compositions of the pregnant solutions and the undissolved residues obtained under both atmospheric and autoclave conditions are similar. This provides additional evidence that atmospheric leaching is the more favorable option for the treatment of ZFR.

The concentrations of iron, copper, and zinc in the pregnant solution after atmospheric leaching were 27.64 g/L, 17.81 g/L, and 1.25 g/L, respectively. This solution’s measured pH has a negative value of −0.05, demonstrating a highly acidic medium. Lead and silver were found to be fully concentrated in the undissolved residue, with slightly higher concentrations observed in the residue from autoclave leaching. Lead is the predominant element in the undissolved residues.

**Figure 6:**
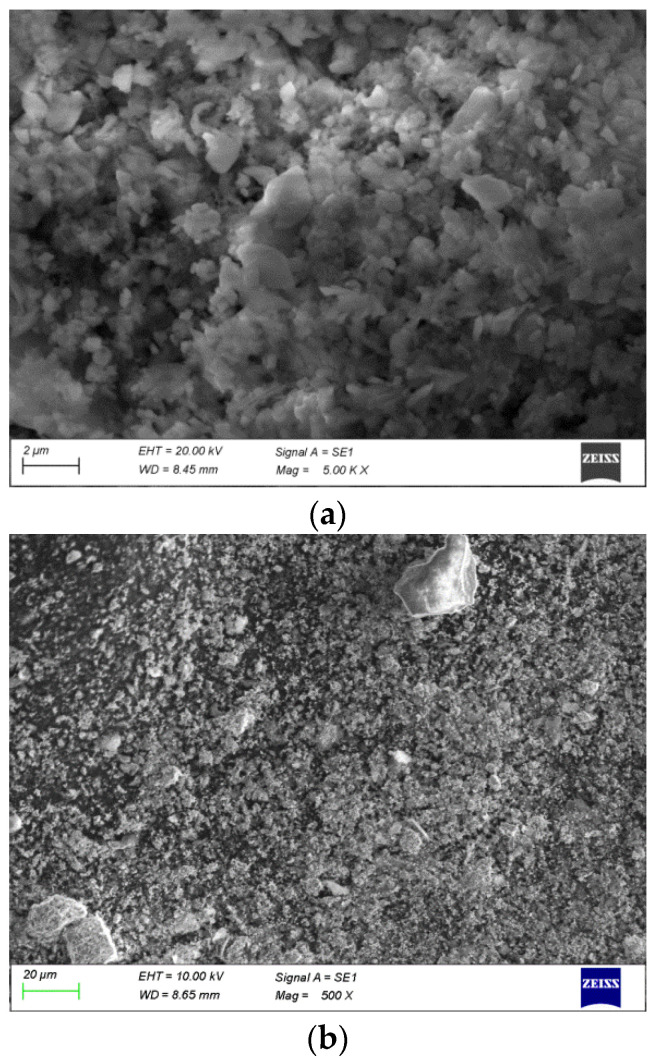
SEM micrographs of the Pb cake. (**a**) Atmospheric leaching; (**b**) autoclave leaching.

The phase composition, morphology, and elemental distribution of the undissolved residues were characterized by XRD and SEM-EDX analyses. Figure 6 presents SEM micrographs of the lead cake obtained after atmospheric (a) and autoclave leaching (b), respectively.

A Map Sum Spectrum of selected areas of the lead residue, given in Table 3 and Figure 6, showed that in addition to lead, there are elevated levels of silicon, iron, and sulfur. The compositions of the analyzed regions are similar.

**Figure 7:**
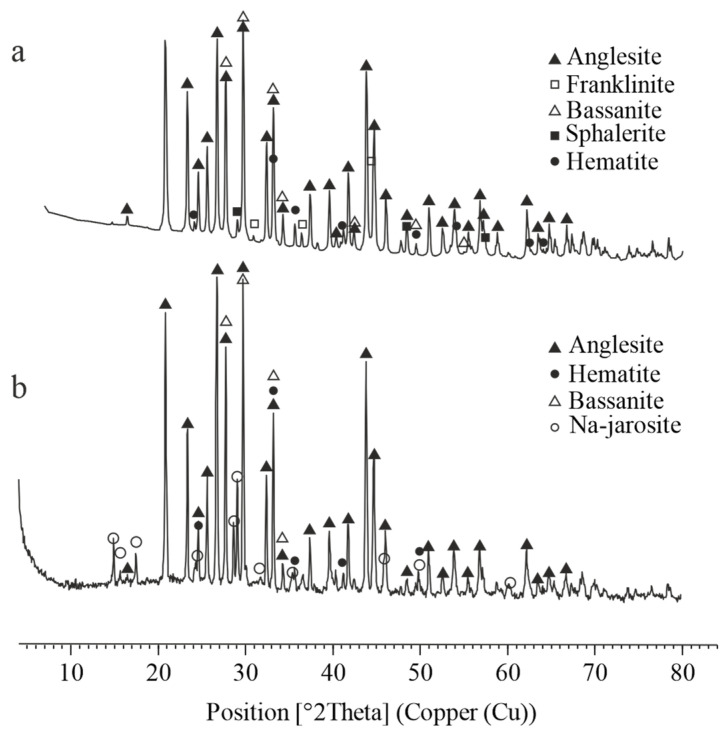
XRD pattern of the lead cake, obtained upon atmospheric and autoclave leaching of ZFR. (**a**) Atmospheric leaching; (**b**) autoclave leaching.

The XRD patterns shown in Figure 7 reveal that the crystalline phases present in the lead residue after atmospheric leaching include anglesite (PbSO_4_), bassanite (CaSO_4_·0.5H_2_O), franklinite (ZnFe_2_O_4_), hematite (Fe_2_O_3_), and sphalerite (ZnS). In contrast, the lead residue obtained after autoclave leaching contains anglesite (PbSO_4_), bassanite (CaSO_4_·0.5H_2_O), sodium jarosite (NaFe_3_(SO_4_)_2_(OH)_6_), and hematite (Fe_2_O_3_).

The lead cake contains 1.13% Zn, which, based on the XRD analysis, is primarily present as franklinite and sphalerite—phases that remained undissolved under the applied atmospheric leaching conditions. Similarly, the iron is present as franklinite and hematite, both of which also did not dissolve due to their low solubility under these conditions.

Franklinite and sphalerite were not identified by XRD in the lead cake obtained after autoclave leaching of the ZFR, most likely because they dissolved at the high temperature and pressure. In this case, some of the iron precipitated as sodium jarosite. XRD analysis revealed the presence of hematite, which may have persisted as an undissolved phase or formed through secondary precipitation during the leaching process.

### 3.2. Precipitation of Hematite

Hematite (Fe_2_O_3_) is a widely occurring iron oxide with diverse industrial applications, including iron and steel production, as a pigment, and in catalytic processes. In conventional zinc-industry practice, hematite precipitation involves an initial reduction in ferric ions (Fe^3+^) to ferrous ions (Fe^2+^), followed by neutralization. The resulting ferrous solution is then subjected to oxidative hydrothermal treatment at elevated temperatures (170–200 °C), where oxygen is introduced to oxidize Fe^2+^ back to Fe^3+^, which subsequently hydrolyzes to form hematite [25,26].

From a theoretical standpoint, hematite precipitation is governed by the hydrolysis of trivalent iron, as described by the following reaction [27]:

2Fe^3+^ + H_2_O → Fe_2_O_3_(s)↓ + 6H^+^ (1)

This reaction leads to acid generation, thereby increasing the acidity of the solution during the precipitation process. Under certain conditions, instead of direct hematite formation, metastable iron hydroxysulfate phases—such as hydronium jarosite and basic ferric sulfate—may precipitate. These phases are pH-dependent and typically transform into hematite upon prolonged hydrothermal treatment or increased temperature, following the sequence [28]:

(2) $$\mathrm{Hydronium}\;\mathrm{jarosite}\;\overset{150\;{}^{\circ}C}{\to }\;\mathrm{Basic}\;\mathrm{ferric}\;\mathrm{sulfate}\;\overset{200\;{}^{\circ}C}{\to }\;\mathrm{Hematite}$$

In the present study, a method for direct hematite precipitation from ferric sulfate solutions was investigated, omitting the reduction step commonly used in industrial processes. Analysis confirmed that the concentration of ferrous iron (Fe^2+^) remained below 100 mg/L, indicating that the iron present was predominantly in the trivalent form. The ferric sulfate solutions were initially neutralized from pH = −0.05 to pH 1.5 using milk of lime. After removal of the resulting gypsum phase, the solutions underwent hydrothermal treatment to facilitate hematite formation.

The effects of temperature (ranging from 150 to 200 °C) and the addition of hematite seed material were systematically studied to evaluate their influence on the degree of iron precipitation; the morphology and crystallinity of the resulting precipitate and the incorporation of impurities were analyzed.

#### Effect of Temperature and Seed Concentration

**Figure 8:**
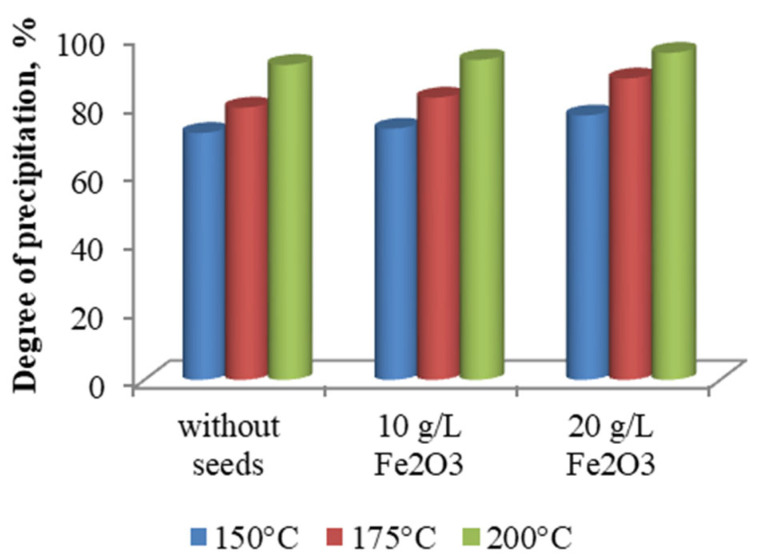
Effect of temperature and seed dosage on the degree of iron precipitation.

The hydrothermal precipitation of iron was investigated at temperatures of 150, 175, and 200 °C, at a solution pH of 1.5, an initial iron concentration of 17.7 g/L, and a reaction time of 2 h. The experimental results, presented in Figure 8, indicate a clear trend of increasing iron precipitation efficiency with rising temperature. In the absence of hematite seed material, the precipitation degree increased from 71.75% at 150 °C to 91.52% at 200 °C. This enhancement is attributed to the accelerated kinetics of ferric hydrolysis and nucleation processes at elevated temperatures, which favor the formation of well-crystallized hematite phases.

**Figure 9:**
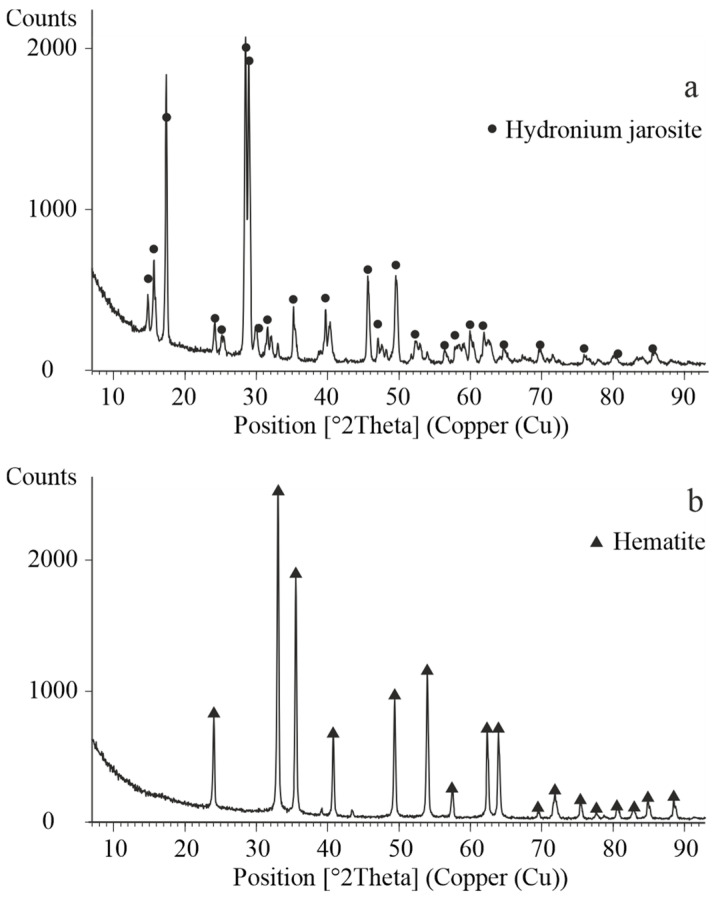
XRD pattern of the obtained precipitate. (**a**) Without seeds; (**b**) in the presence of 20 g/L seeds.

The precipitate obtained at 150 °C was characterized by XRD analysis. As shown in Figure 9a, the only crystalline phase identified was jarosite, indicating that under these conditions and in the absence of hematite seeds, the hydrolysis of ferric iron favors the formation of intermediate iron hydroxysulfate compounds rather than hematite.

The effect of temperature in the presence of hematite seed material at concentrations of 10 and 20 g/L is also illustrated in Figure 8. The experimental data show that the degree of iron precipitation increased from 76.83% to 95.02% as the temperature rose within the studied range, when 20 g/L of hematite seeds were used. Reducing the seed concentration to 10 g/L resulted in a decrease in precipitation efficiency by approximately 3–4%, highlighting the catalytic role of hematite seeds in promoting nucleation and accelerating crystallization processes.

**Figure 10:**
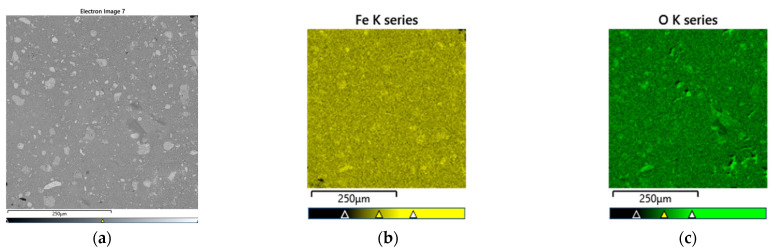
SEM micrographs and EDS element mapping of hematite. (**a**) SEM image; (**b**) Fe distribution; (**c**) O distribution.

The precipitate formed at a temperature of 200 °C in the presence of 20 g/L hematite seeds was subjected to detailed characterization using XRD analysis (Figure 9b). In addition, the morphology of the precipitate was investigated by SEM. Representative SEM images, along with elemental mapping for iron and oxygen, are shown in Figure 10, and the corresponding chemical composition of a selected particle is presented in Table 4.

The results show that iron and oxygen are the predominant elements, confirming the hematite phase. Minor impurities detected include sulfur, silicon, lead, and calcium, present in trace amounts. The low concentration of impurities suggests the high purity of the obtained hematite, indicating its potential suitability for commercial applications.

The solution obtained from sulfuric acid leaching, after purification from iron and other impurities, can be subjected to zinc electrowinning. The spent electrolyte generated in this step can be recycled back to the leaching stage, thereby enhancing resource efficiency and minimizing waste.

### 3.3. Chloride Leaching of Pb Cake

As indicated by the analyses of the lead cake obtained after sulfuric acid leaching of ZFR, lead is present predominantly in the form of anglesite (PbSO_4_), with a concentration of approximately 27%. According to the literature [29,30,31,32], the most suitable method for extracting lead from this residue is chloride leaching, during which the following reactions take place:

PbSO_4_(s) + 2Cl^−^ → PbCl_2_(s) + SO_4_^2−^ (3)

PbCl_2_(s) + Cl^−^ → PbCl_3_ (4)

PbCl_3_^−^ + Cl^−^ → PbCl_4_^2−^ (5)

The influence of several process parameters on lead extraction efficiency was investigated, along with the leaching behavior of other metals such as silver, zinc, iron, and copper.

#### 3.3.1. Effect of NaCl Concentration

**Figure 11:**
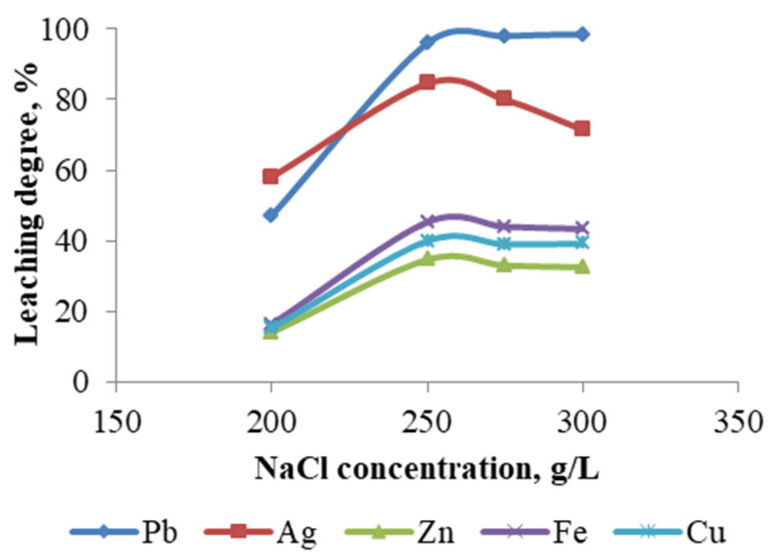
Degree of metal leaching depending on NaCl concentration.

The impact of varying NaCl concentrations (200–300 g/L) on the metal leaching was examined under constant parameters. The constant parameters included HCl concentration (1 M), temperature (60 °C), pulp density (10%), and duration (1 h). The recovery of the metals under consideration increased with increasing NaCl concentration above 100 g/L (Figure 11), with some showing a higher rate of increase (lead and silver) and others a lower rate.

The obtained results indicate that the concentration of chloride ions is a significant parameter influencing lead and silver recovery.

With increasing chloride ion concentration, the insoluble PbCl_2_ is converted into soluble complexes such as PbCl_3_^−^ and PbCl_4_^2−^. The maximum lead extraction efficiency of 98.51% was achieved at a NaCl concentration of 300 g/L. At the same NaCl concentration, the extraction efficiency of silver was reduced to 71.5%. The leaching degrees for the remaining metals, including copper, zinc, and iron, were found to be considerably lower.

It should be noted that in some of the experiments, secondary precipitation of Pb was observed when the temperature was lowered or when the pulp was washed during filtration, since the solubility of PbCl_2_ was significantly reduced when the temperature was lowered and when the solution was diluted.

#### 3.3.2. Effect of HCl Concentration

**Figure 12:**
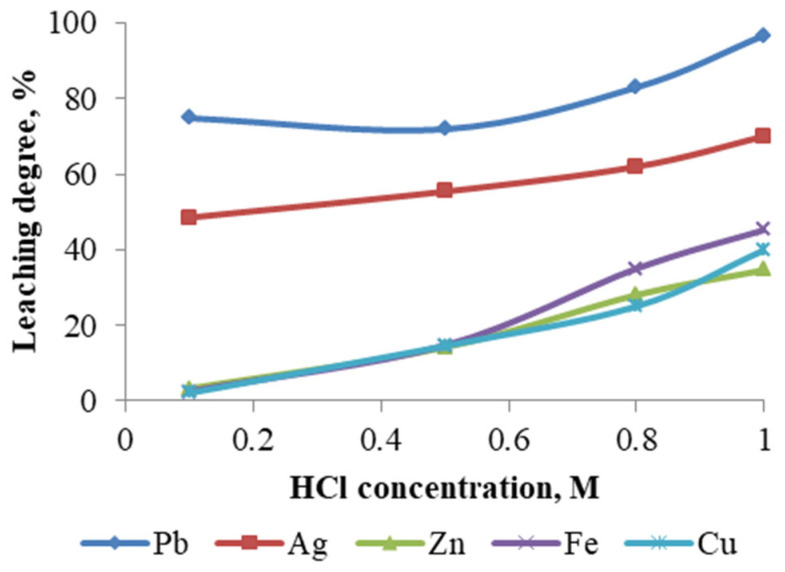
Degree of metal leaching depending on HCl concentration.

Experiments were conducted to evaluate the effect of HCl concentration, within the range of 0.1–1 M, on the metal leaching efficiency (Figure 12), while maintaining the following parameters constant: temperature—60 °C; leaching duration—1 h; NaCl concentration—250 g/L; and pulp density—10%.

As the acid concentration is increased, a corresponding increase in the mass reduction rate of the cake is observed. This, in turn, leads to an elevated metal recovery rate. Utilizing a 1 M HCl solution, a maximum lead recovery of 96.70% was attained, accompanied by a 55% reduction in the initial material mass. It is important to note that increasing the acid concentration above 1 M will result in production solutions with very low pH values, which complicates the subsequent extraction process of lead and silver from solution.

#### 3.3.3. Effect of Temperature

**Figure 13:**
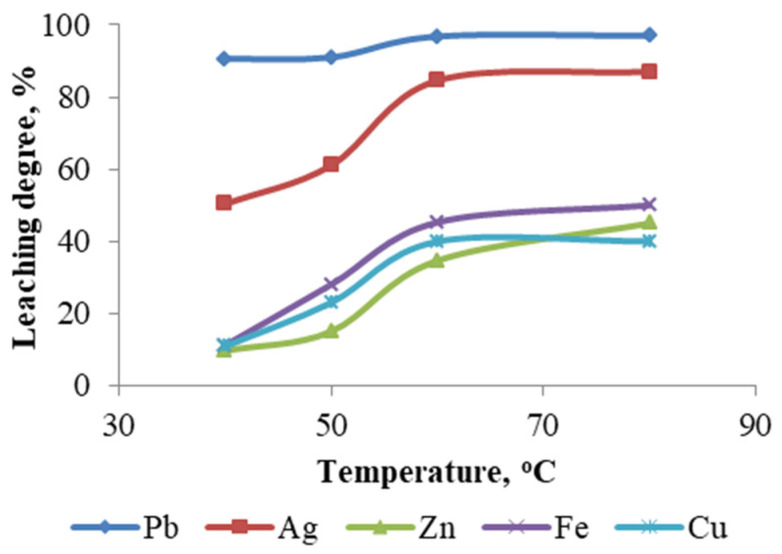
Degree of metal leaching depending on temperature.

To investigate the effect of temperature on metal leaching efficiency, four experiments were conducted at 40, 50, 60, and 80 °C, while maintaining the following parameters constant: NaCl concentration—250 g/L; HCl concentration—1 M; pulp density—10%; and leaching duration—1 h. The results of these experiments are presented in Figure 13.

As demonstrated in the figure, the temperature has a significant impact on the solubility of metals, including Ag, Cu, Zn, and Fe. However, the extraction rate of Pb remains relatively constant regardless of temperature variations.

#### 3.3.4. Effect of Time

A series of experiments were conducted in which the duration of leaching was varied while maintaining constant conditions. The experimental setup included a sodium chloride concentration of 250 g/L, a hydrochloric acid concentration of 1 M, a pulp density of 10%, and a temperature of 60 °C.

**Figure 14:**
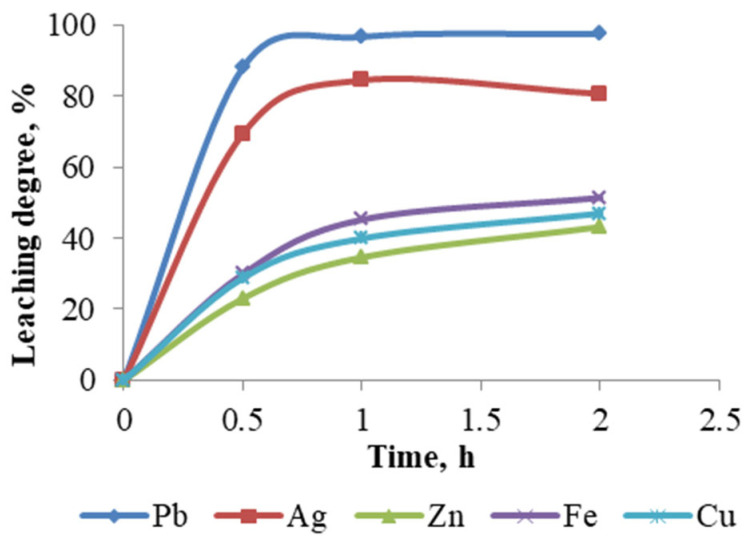
Degree of metal leaching depending on time.

The experimental results (Figure 14) demonstrate that during the initial hour, there is significant dissolution of the lead cake, followed by a slight increase in the rate of extraction of copper, iron, and zinc. In contrast, the extraction of lead remains relatively unchanged, while that of silver leaching degree has decreased slightly. The maximum recoveries of lead and silver under these conditions are 97.62% and 84.55%, respectively.

#### 3.3.5. Effect of Solid–Liquid Ratio

**Figure 15:**
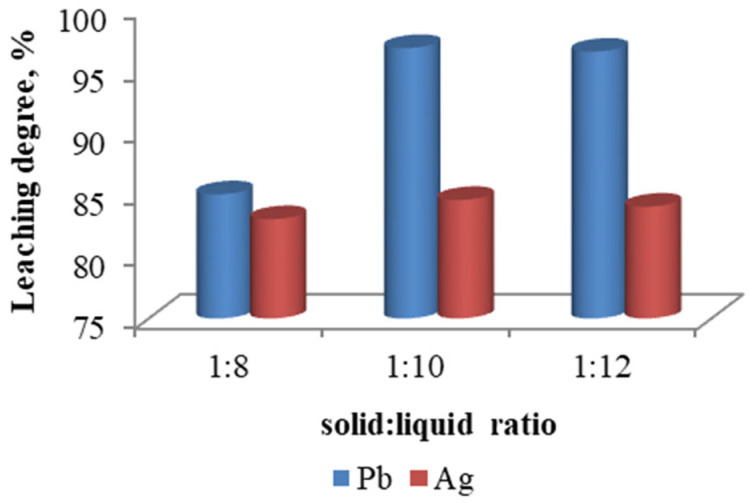
Degree of metal leaching depending on solid–liquid ratio.

The effect of pulp density on the recovery rate of lead and silver was investigated, and the results are presented in Figure 15. As the solid–liquid ratio increases above 1:10, the leaching degree of lead and silver remains constant.

In accordance with the conducted experiments and the obtained results, the optimal conditions for the chloride leaching of the lead cake can be determined:

- NaCl concentration—250 g/L;
- HCl concentration—1 M;
- Process duration—1 h;
- Temperature—60 °C.

Consequently, a production solution and insoluble residue with chemical compositions outlined in Table 5 were obtained at the specified optimal conditions.

It has been demonstrated that, within the established optimal parameters for chloride leaching, a mass reduction of 54.75% was reached for the initial lead cake. Furthermore, the leaching rates for lead and silver were found to be 96.79% and 84.55%, respectively.

**Figure 16:**
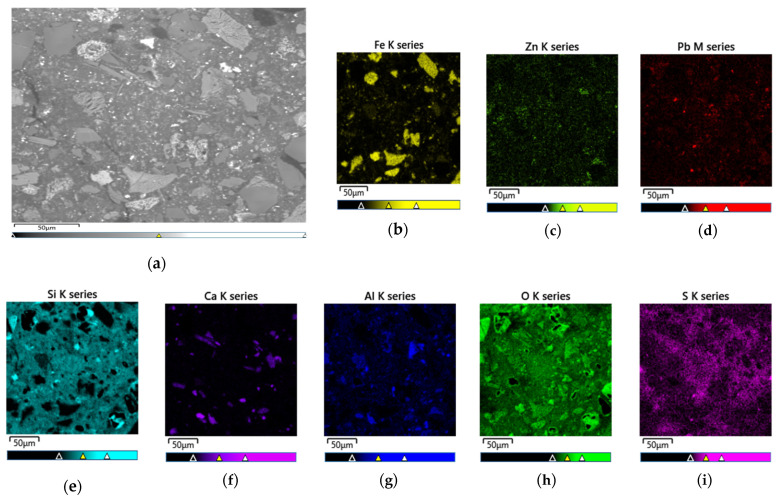
SEM micrograph of insoluble residue, obtained upon the chloride leaching of Pb cake (**a**) and EDS elemental mapping in the insoluble residue after chloride leaching of Pb cake: (**b**) Fe; (**c**) Zn; (**d**) Pb; (**e**) Si; (**f**) Ca; (**g**) Al; (**h**) O; (**i**) S.

After chloride leaching, the solid residue was examined using SEM–EDS. The SEM image (Figure 16a) revealed a heterogeneous morphology, with angular grains (likely ferritic phases), fibrous PbCl_2_/PbSO_4_ structures, and a porous matrix possibly composed of silicate minerals.

EDS analysis (Table 6) showed that oxygen (44.33%), silicon (25.57%), and iron (14.68%) were dominant, indicating silicate and iron-oxide phases.

**Figure 17:**
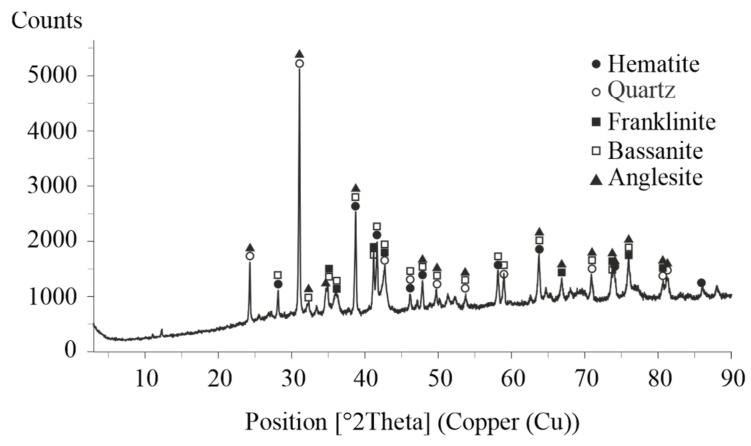
XRD pattern of the insoluble residue, obtained upon chloride leaching of the Pb cake.

XRD (Figure 17) identified the crystalline phases in the residue as hematite (Fe_2_O_3_), quartz (SiO_2_), franklinite (ZnFe_2_O_4_), albite (NaAlSi_3_O_8_), and anglesite (PbSO_4_). The detection of PbSO_4_ in the solid residue suggests incomplete extraction of lead during the chloride leaching process. This observation is further corroborated by the results of ICP analysis, as presented in Table 6.

### 3.4. Cementation of Lead and Silver from Chloride Leachate

Based on previous experience [33], an effective method for recovering lead and silver from the chloride leachate is the cementation process using zinc powder. This method allows for the selective precipitation of target metals through redox reactions, exploiting the more negative standard electrode potential of zinc.

In the conducted experiment, the production solution was first neutralized to a pH of 2.4 using a 10% NaOH solution at room temperature. Cementation was then performed by adding zinc powder in a 200% stoichiometric excess relative to the amount required to reduce all soluble lead and silver. The reaction proceeded for 1 h under constant stirring.

The chemical composition of the resulting cementation precipitate is summarized in Table 7, confirming efficient selective recovery of lead and silver, with lead content reaching 84.75% and silver 0.1740%. The low levels of residual zinc, iron, and copper indicate effective separation and minimal contamination.

### 3.5. Hydrometallurgical Process Scheme for ZFR Treatment

**Figure 18:**
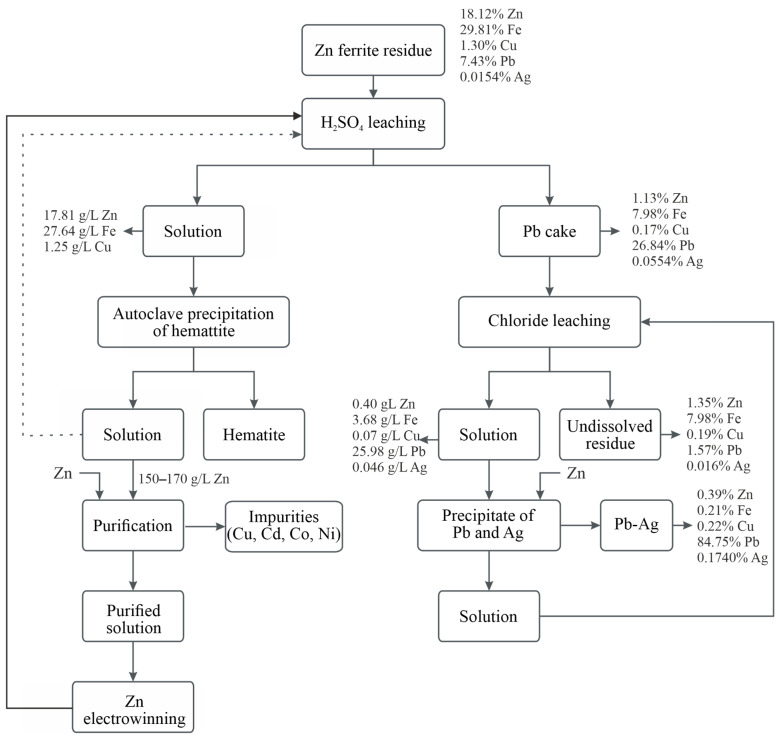
Hydrometallurgical process flowchart for the treatment of ZFR.

Figure 18 presents the overall technological scheme for the hydrometallurgical treatment of ZFR. It visualizes the sequence of the main stages through which the primary valuable metals—zinc, iron, lead, and silver—are extracted and separated.

The solution obtained from sulfuric acid leaching, which initially contains relatively low concentrations of zinc, can be reused in successive leaching cycles of fresh portions of ZFR. This approach gradually increases the zinc concentration in the solution, typically up to 150–170 g/L. As the solution is reused and its metal content increases, the concentrations of impurity elements also rise. Once sufficient zinc concentration is achieved, solution purification (such as cementation using zinc powder) should be carried out to remove impurities like copper, cadmium, cobalt, and nickel. After purification, the solution can be subjected to zinc electrowinning. The resulting spent electrolyte from the electrowinning process can then be recycled back to the leaching stage, thus establishing a closed-loop process that enhances zinc recovery efficiency and minimizes waste.

The solution remaining after the cementation of lead and silver can be reused for the leaching of additional portions of lead cake, thus contributing to a more sustainable and cost-effective process.

The entire scheme demonstrates an efficient, stepwise approach for the recovery of valuable components from ZFR. It is characterized by selectivity of the processes, good separation of the fractions, and potential for application under industrial conditions.

It is important to note that the final insoluble residue obtained after chloride leaching still contains traces of non-ferrous and precious metals, particularly lead, rendering it a hazardous material. We have some ideas for potential approaches to reduce the concentrations of lead and silver in the final residue; however, we are not yet certain that these will yield significant improvements. This underscores the need for further research focused on either enhanced metal recovery or the safe disposal/stabilization of the residue to meet environmental regulations.

## 4. Conclusions

Sulfuric acid leaching under atmospheric conditions (90 °C, 200 g/L H_2_SO_4_) provides a high extraction degree of zinc (98.3%), copper (96.4%), and iron (92.72%) from the ZFR, with temperature and acid concentration identified as determining factors for process efficiency.

Autoclave processing of the production solution leads to effective precipitation of iron in the form of hematite, especially when hematite seeds are used. The resulting product has high purity and low impurity content.

Chloride leaching of the lead cake shows that under optimal conditions, high extraction rates of lead (96.79%) and silver (84.55%) are achieved, with a reduction in residue mass by about 55%.

Cementation with zinc powder is an effective method for precipitating lead and silver from the chloride solution. Yields of 98.72% for Pb and 97.27% for Ag were achieved, and the precipitate contains 84.75% Pb and silver 0.174% Ag.

Despite the efficient extraction of the target metals, the residual material after chloride leaching still contains non-ferrous and precious metals, most likely embedded in stable mineral phases. The presence of lead in the residue classifies it as hazardous waste.

The proposed scheme is technologically justified, demonstrates high selectivity, and can be implemented for treating ZFR. Further development is needed for effective treatment of the final insoluble residue.

## Figures and Tables

**Table 1:** Main chemical composition of ZFR (mass fraction, %).

Zn	Fe	Cu	Pb	Ag	Mn	S	Si	Ca	Al
18.12	29.81	1.30	7.43	0.0154	1.84	3.52	3.27	2.21	1.11

**Table 2:** Chemical composition of the products obtained from sulfuric acid leaching of ZFR.

Type of Leaching	Products	Chemical Composition				
		Fe	Zn	Pb	Cu	Ag
Atmospheric leaching	Solution g/L	27.64	17.81	-	1.25	-
	Insoluble residue %	7.98	1.13	26.84	0.17	0.0554
Autoclave leaching	Solution g/L	27.59	17.76	-	1.26	-
	Insoluble residue %	9.12	1.47	30.53	0.168	0.0634

**Table 3:** EDS analysis of Pb cake.

Element	Weight % (Atmospheric Leaching)	Weight % (Autoclave Leaching)
Pb	29.48	25.77
Fe	7.51	9.78
Zn	2.03	1.86
S	5.46	5.42
O	38.59	40.78
Si	12.42	11.15
Al	1.32	1.47
Ca	0.43	1.22
K	0.00	0.66
Na	0.32	0.63
Mg	0.12	0.58
Mn	1.99	0.36
Cu	0.33	0.33

**Table 4:** EDS analysis of hematite.

Element	Weight %
Fe	75.28
O	23.28
S	0.90
Si	0.29
Pb	0.21
Ca	0.04

**Table 5:** Chemical composition of the chloride leaching products.

	Zn	Fe	Cu	Pb	Ag
Insoluble residue, %	1.35	7.98	0.19	1.57	0.016
Solution, g/L	0.40	3.61	0.07	25.98	0.046

**Table 6:** EDS analysis of the residue after chloride leaching.

Element	Weight %
Fe	15.70
Pb	1.50
Zn	4.82
S	1.67
Si	25.57
Al	3.58
O	44.33
Ti	1.02
Mg	0.87
Ca	0.76
Na	0.19

**Table 7:** Chemical composition of the cementation precipitate (mass fraction, %).

Zn	Fe	Cu	Pb	Ag
0.39	0.21	0.22	84.75	0.1740

## Data Availability

The original contributions presented in this study are included in the article. Further inquiries can be directed to the corresponding authors.

## Abbreviations

The following abbreviations are used in this manuscript:

ZFR	Zinc Ferrite Residue
SEM	Scanning electron microscope
EDS	Energy Dispersive X-ray Spectroscopy
ICP-OES	Inductively Coupled Plasma Optical Emission Spectroscopy
XRD	X-ray diffraction
